# Enhancement of membrane-type 1-matrix metalloproteinase (MT1-MMP) production and sequential activation of progelatinase A on human squamous carcinoma cells co-cultured with human dermal fibroblasts

**DOI:** 10.1038/sj.bjc.6690477

**Published:** 1999-06

**Authors:** T Sato, M Iwai, T Sakai, H Sato, M Seiki, Y Mori, A Ito

**Affiliations:** Department of Biochemistry, School of Pharmacy, Tokyo University of Pharmacy and Life Science, Hachioji, Tokyo, 192-0392, Japan; Department of Molecular Virology and Oncology, Cancer Research Institute, School of Medicine, Kanazawa, Ishikawa, 920-0934, Japan; Department of Tumor Cell Research, Institute of Medical Science, University of Tokyo, Minato-ku, Tokyo, 108-0071, Japan

**Keywords:** membrane type-1 matrix metalloproteinase, progelatinase A, co-culture, human squamous carcinoma cells, human dermal fibroblasts

## Abstract

Matrix metalloproteinase 2 (MMP-2)/gelatinase A plays an important role in tumour invasion and metastasis. Since MMP-2 is secreted as an inactive form (proMMP-2) from tumour and neighbouring stroma cells, the activation process is necessary to express the enzymic activity for degradation of extracellular matrix components. We herein reported that the activation of proMMP-2 was induced in human squamous carcinoma cells co-cultured with normal human dermal fibroblasts. When A431 cells were co-cultured with human fibroblasts at various cell ratios, 72-kDa proMMP-2 was converted to a 62-kDa active form through the appearance of a 64-kDa intermediate. The activation of proMMP-2 by co-culture was also observed in other carcinoma cell lines, HSC-4 and SAS, but not in normal human keratinocytes. We characterized by in vitro invasion assay that A431 cells in co-culture preferentially invaded through Matrigel and the increased invasive activity was inhibited by exogenously adding tissue inhibitor of metalloproteinases 2. The augmented proMMP-2 activation by co-culture was achieved by the increase in membrane type 1-MMP (MT1-MMP) production along with that of its mRNA level. The predominant appearance of MT1-MMP was immunologically observed in A431 cells, but not human fibroblasts of the co-culture. Furthermore, epidermal growth factor (EGF) enhanced the co-culture-mediated proMMP-2 activation by increasing the production and gene expression of MT1-MMP, and thereby tumour invasive activity was further augmented. These results suggest that the cell–cell contact between carcinoma cells and normal fibroblasts enhances the production of MT1-MMP followed by sequential activation of proMMP-2 on the tumour cell surface, which may be closely implicated in tumour invasion in vivo. © 1999 Cancer Research Campaign


British Journal of Cancer (1999) **80**(8), 1137–1143
© 1999 Cancer Research Campaign
Article no. bjoc.1999.0477

### Enhancement of membrane-type 1-matrix metalloproteinase (MT1-MMP) production and sequential activation of progelatinase A on human squamous carcinoma cells co-cultured with human dermal fibroblasts

**T Sato[1], M Iwai[1], T Sakai[1], H Sato[2], M Seiki[3], Y Mori[1] and A Ito[1]**

[1]Department of Biochemistry, School of Pharmacy, Tokyo University of Pharmacy and Life Science, 1432-1 Horinouchi, Hachioji, Tokyo 192-0392, Japan; [2]Department of Molecular Virology and Oncology, Cancer Research Institute, School of Medicine, Kanazawa University, Kanazawa, Ishikawa 920-0934, Japan; [3]Department of Tumor Cell Research, Institute of Medical Science, University of Tokyo, Shirokanedai, Minato-ku, Tokyo 108-0071, Japan

**Summary** Matrix metalloproteinase 2 (MMP-2)/gelatinase A plays an important role in tumour invasion and metastasis. Since MMP-2 is secreted as an inactive form (proMMP-2) from tumour and neighbouring stroma cells, the activation process is necessary to express the enzymic activity for degradation of extracellular matrix components. We herein reported that the activation of proMMP-2 was induced in human squamous carcinoma cells co-cultured with normal human dermal fibroblasts. When A431 cells were co-cultured with human fibroblasts at various cell ratios, 72-kDa proMMP-2 was converted to a 62-kDa active form through the appearance of a 64-kDa intermediate. The activation of proMMP-2 by co-culture was also observed in other carcinoma cell lines, HSC-4 and SAS, but not in normal human keratinocytes. We characterized by in vitro invasion assay that A431 cells in co-culture preferentially invaded through Matrigel and the increased invasive activity was inhibited by exogenously adding tissue inhibitor of metalloproteinases 2. The augmented proMMP-2 activation by co-culture was achieved by the increase in membrane type 1-MMP (MT1-MMP) production along with that of its mRNA level. The predominant appearance of MT1-MMP was immunologically observed in A431 cells, but not human fibroblasts of the co-culture. Furthermore, epidermal growth factor (EGF) enhanced the co-culture-mediated proMMP-2 activation by increasing the production and gene expression of MT1-MMP, and thereby tumour invasive activity was further augmented. These results suggest that the cell–cell contact between carcinoma cells and normal fibroblasts enhances the production of MT1-MMP followed by sequential activation of proMMP-2 on the tumour cell surface, which may be closely implicated in tumour invasion in vivo.

**Keywords:** membrane type-1 matrix metalloproteinase; progelatinase A; co-culture; human squamous carcinoma cells; human dermal fibroblasts

The metastatic progression of malignant tumour requires the proteolytic degradation of extracellular matrix components such as type IV collagen, laminin and proteoglycans. Matrix metalloproteinases (MMPs) play important roles in the extracellular matrix degradation; especially the expression of MMP-2/gelatinase A (72-kDa type IV collagenase) correlates closely with the invasive and metastatic phenotype of tumour cell in vivo (Pyke et al, 1992; Stearns and Wang, 1993; Stetler-Stevenson et al, 1993). MMP-2 is biosynthesized as a proenzyme (proMMP-2) as well as other MMPs, and the proteolytic cleavage of propeptide in the zymogen is essential to express enzymic activity in vitro (Okada et al, 1990; Birkedal-Hansen et al, 1993; Itoh et al, 1995). Concerning the activation of proMMPs in vivo, MMP-3/stromelysin-1 is identified as an endogenous activator for proMMP-1/interstitial procollagenase and proMMP-9/progelatinase B (Ito and Nagase, 1988; Ito et al, 1991; Ogata et al, 1992), but not for proMMP-2 (Okada et al, 1990). Baramova et al (1997) and Mazzieri et al (1997) reported the involvement of plasminogen activator/plasmin system in proMMP-2 activation. Recently, membrane type-MMPs, MT1-MMP (Sato et al, 1994), MT2-MMP (Will and Hinzmann, 1995), MT3-MMP (Takino et al, 1995) and MT4-MMP (Puente et al, 1996) have been discovered and reported to specifically activate proMMP-2 on the cell surface. Furthermore, increased expression of MT1-MMP has been shown to correlate with proMMP-2 activation in human malignant tumours in vivo (Yamamoto et al, 1996; Ueno et al, 1997). Thus, MT1-MMP is considered to be a specific in vivo activator for proMMP-2.

The activation of proMMP-2 is induced by concanavalin A and 12-*O*-tetradecanoylphorbol 13-acetate in human fibroblasts and human fibrosarcoma HT-1080 cells respectively (Ward et al, 1991; Strongin et al, 1993). Lohi et al (1996) also reported that 12-*O*-tetradecanoylphorbol 13-acetate and concanavalin A significantly induce the expression of MT1-MMP in HT-1080 cells and human embryonic lung fibroblasts. On the other hand, Seltzer et al (1994) reported that $\beta_1$ integrin receptor, possibly $\alpha_2\beta_1$ augments the conversion of 72-kDa proMMP-2 to 62-kDa active form in human fibroblasts cultured in collagen lattices. In addition, calcium ionophores inhibit the phorbol ester-induced proMMP-2 activation

*Received 24 June 1998*
*Revised 24 November 1998*
*Accepted 6 January 1999*

*Correspondence to:* T Sato in HT-1080 cells (Lohi and Keski-Oja, 1995) and calmodulin antagonists accelerate the proMMP-2 activation in human fibroblasts (Ito et al, 1998), but possible in vivo cellular mechanisms controlling the production of MT-MMPs and the activation of proMMP-2 remain unclear.

In the present study, we demonstrated that the activation of proMMP-2 was induced by co-culture of human carcinoma cells and normal human fibroblasts and thereby in vitro tumour invasive activity was accelerated. The augmentation of proMMP-2 activation by the co-culture was associated with the predominant increase in MT1-MMP production on carcinoma cells.

### MATERIALS AND METHODS

### Cell lines and cell culture conditions

Human epidermoid squamous carcinoma cell line, A431 (RIKEN Cell Bank, Ibaraki, Japan), human oral squamous carcinoma cell lines, HSC-4 and SAS (Health Science Research Resources Bank, Osaka, Japan) and normal human dermal fibroblasts (Sato et al, 1997) were cultured in Dulbecco's modified Eagle medium (DMEM) (Life Technologies, Inc., Grand Island, NY, USA) supplemented with 10% (v/v) fetal bovine serum (BioWhittaker, Walkersville, MD, USA), 200 units $ml^{-1}$ of penicillin and 200 μg $ml^{-1}$ of streptomycin until confluence. Human epidermal keratinocytes (Kyokuto Pharmaceutical Industrial Co., Tokyo, Japan) were cultured as described previously (Sato et al, 1997). For co-culture experiments, human dermal fibroblasts were first seeded at $3.5 \times 10^4$ cells $cm^{-2}$ in 24-well culture plates or 100-mm diameter dishes and grown to confluence. The cells from three wells or dishes were removed with 0.25% (w/v) trypsin – 0.02% (w/v) EDTA/$Ca^{2+}$ and $Mg^{2+}$-free phosphate-buffered saline (PBS (–)) and the number of cells were determined. Based on the number of cells, carcinoma cells or keratinocytes as normal epithelial cells were placed on the confluent monolayer culture of fibroblasts at various cell ratios and allowed to attach for 24 h. After 24-h incubation, the cells were washed with the serum-free culture medium once and then treated with or without recombinant human epidermal growth factor (EGF) (Genzyme, Cambridge, MA, USA) in the same culture medium. In this series of experiments, A431 cells, human dermal fibroblasts and human epidermal keratinocytes were used at the 11th–24th, 10th–22nd and 4th–8th passage levels respectively.

### Gelatin zymography

Gelatinolytic activity in culture media from the monolayer or the co-culture was detected by gelatin zymography as described previously (Takahashi et al, 1991). Briefly, an aliquot (10 μl) of the culture medium was subjected to sodium dodecyl sulphate-polyacrylamide gel electrophoresis (SDS-PAGE) using a 10% (w/v) acrylamide gel containing 0.6 mg $ml^{-1}$ of gelatin (DIFCO Laboratories, Detroit, MI, USA). The gel was washed with washing buffer (50 mM Tris–HCl (pH 7.5), 0.15 M sodium chloride, 10 mM calcium chloride, 1 μM zinc chloride, 0.1% (v/v) Triton X-100) to remove SDS and then incubated with incubation buffer (50 mM Tris–HCl (pH 7.5), 0.15M sodium chloride, 10 mM calcium chloride, 1 μM zinc chloride) for 6 h at 37°C. After incubation, the gel was stained with Coomassie brilliant blue R-250 and gelatinolytic activity was detected as unstained bands on a blue background.

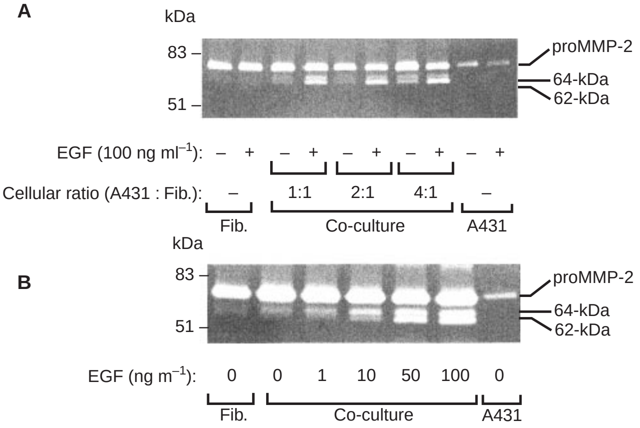

**Figure 1** Augmentation of proMMP-2 activation in co-culture of A431 cells and human dermal fibroblasts. (**A**) Confluent human dermal fibroblasts (Fib.) at the 10th passage were co-cultured with A431 cells at the 18th passage at the indicated cellular ratio for 24 h and then the cells were treated with or without EGF (100 ng $ml^{-1}$) for a further 24 h. In monolayer culture of A431 cells, the number of cells seeded corresponded to that for the cellular ratio of 4. (**B**) A431 cells at the 21st passage were seeded on confluent human dermal fibroblasts (Fib.) at the 14th passage at the cellular ratio of 4 and the co-culture was performed for 24 h. A431 cells were also cultured at the number corresponding to the cellular ratio of 4 for 24 h. Those cells were treated with or without EGF (1–100 ng $ml^{-1}$) for a further 24 h. An aliquot (10 μl) of the harvested culture medium was subjected to gelatin zymography as described in Materials and Methods. Three independent experiments were reproducible and typical data are shown

### Western blot analysis for MT1-MMP

To monitor the production of MT1-MMP, the cells were washed with ice-cold PBS (–) once and then scraped into 50 mM Tris–HCl (pH 7.4), 0.24 M sucrose with a rubber policeman. After centrifugation at 180 ***g*** for 5 min at 4°C, the cells were resuspended in the same buffer, sonicated six times at 4°C for 10 s and then centrifuged at 500 ***g*** for 20 min at 4°C. The supernatant was centrifuged at 100 000 ***g*** for 60 min. The subsequent precipitate was resuspended in 50 mM Tris–HCl (pH 7.4) and then subjected to Western blot analysis for detection of MT1-MMP. We confirmed that MT1-MMP was not detected in the cytosolic fraction. The protein concentration in the samples was determined according to the method of Lowry et al (1951). A part (100 μg) of the precipitate was mixed with SDS-PAGE sample buffer including 2-mercaptoethanol (Laemmli, 1970) and then run on 10% (w/v) acrylamide gel. The proteins separated on the gel were electrotransferred onto a nitrocellulose membrane. The membrane was reacted with anti-MT1-MMP monoclonal antibody (114-1F2), which was then complexed with alkaline phosphatase conjugated rabbit anti-(mouse IgG)IgG. Immunoreactive MT1-MMP was visualized with 5-bromo-4-chloro-3-indolyl phosphate and nitro blue tetrazolium as described previously (Ito et al, 1998). The relative amounts of MT1-MMP were quantified by densitometric scanning.

### RNA isolation and Northern blot analysis

Total RNA was isolated from the co-culture of A431 cells and fibroblasts or each monolayer culture by using ISOGEN (Nippon Gene Co., Toyama, Japan) according to the protocol recommended by the manufacturer. Isolated RNA (15 μg) was denatured and

*© 1999 Cancer Research Campaign*

electrophoresed on 1.0% (w/v) agarose gel and then transferred onto a nylon membrane (GeneScreen, DuPont, Boston, MA, USA). The membrane was hybridized with cDNA probes for MT1-MMP (1.5 kb) and human glyceraldehyde-3 phosphate dehydrogenase (GAPDH) (1.1 kb, CLONTECH Laboratories, Inc., Palo Alto, CA, USA) as described previously (Ito et al, 1998). The relative amounts of steady-state MT1-MMP mRNA were quantified by densitometric scanning and indicated after correction for the GAPDH mRNA level.

### Tumour invasion assay

Tumour invasion assay was performed using Matrigel Invasion Chamber (Nippon Becton Dickinson Co. Ltd., Tokyo, Japan). A431 cells and human fibroblasts were labelled with or without 5- and 6-carboxyfluorescein diacetate succinimidyl ester (CFSE) (Dojindo Laboratories, Kumamoto, Japan) (Bronner-Fraser, 1985) in 0.1% (w/v) bovine serum albumin–DMEM for 2 h. CFSE-labelled human dermal fibroblasts and A431 cells were added into the inserts together with CFSE-unlabelled A431 cells and human dermal fibroblasts, respectively, with $4 \times 10^4$ cells (for A431 cells) and $1 \times 10^4$ cells (for human fibroblasts). Following cell adhesion for 1 h, the cells were treated with or without EGF (100 ng $ml^{-1}$) in the presence or the absence of recombinant human tissue inhibitor of metalloproteinases 2 (TIMP-2) (a generous gift from Dr H Nagase, University of Kansas Medical Center). After 48 h incubation, cells on the upper surface of the membrane were wiped with a cotton swab and the number of fluoresced cells on the lower side in ten randomly chosen areas per membrane was counted under a fluorescent microscope at 200-fold magnification.

### Immunostaining of MT1-MMP

Cultured A431 cells and/or human fibroblasts on chamber slides (Nunc Inc., Naperville, IL, USA) were fixed for 1 h in freshly prepared 4% (v/v) formaldehyde–PBS (–) and washed twice for 5 min with PBS (–). The fixed cells were treated with 0.3% (v/v) hydrogen peroxide in MeOH for 1 h to quench endogenous peroxidase, washed again with PBS (–) and then treated with 1.5% (v/v) normal horse serum diluted in PBS (–) for 1 h to block non-specific binding of the antibody. The cells were incubated with anti-MT1-MMP monoclonal antibody (5 μg $ml^{-1}$) overnight at 4°C. Non-immune mouse IgG was utilized as a negative control for the primary antibody. The cells were washed with PBS (–), stained by Vectastain ABC Kit (Vector Laboratories, Inc., Burlingame, CA, USA).

### Statistical analysis

Data were analysed by Student's *t*-test; $P < 0.05$ was considered statistically significant.

### RESULTS

### Activation of proMMP-2 by co-culture of A431 cells and human fibroblasts

Human squamous carcinoma A431 cells were placed on a monolayer of confluent human dermal fibroblasts and then gelatin zymography of the harvested culture media was performed. Figure 1A showed that fibroblasts spontaneously produced proMMP-2 but A431 cells did so poorly. The production of proMMP-2 was unchanged by the co-culture as compared to that for the monolayer culture of fibroblasts. When the cellular ratio of A431 cells to fibroblasts was increased to 4, both 64-kDa intermediate and 62-kDa active form of MMP-2 were increased. The proMMP-2 activation was observed with $^1/_4$ cellular ratio of A431 (data not shown) and significantly with 1-4 to human fibroblasts. These results indicated that the cell–cell interaction of A431 cells and fibroblasts resulted in the activation of proMMP-2, and that the augmented proMMP-2 activation was dependent on the increase of tumour cell number in co-culture.

EGF is known to participate in the tumorigenesis by augmenting proliferation of carcinoma cells because those cells abundantly express EGF receptor (Ozawa et al, 1987; Singletary et al, 1987). In addition, Shima et al (1993) reported that the production of proMMP-9 in oesophageal squamous carcinoma cells is augmented by EGF. We therefore examined whether EGF could modify the activation of proMMP-2 induced by co-culture of A431 cells and fibroblasts. As shown in Figure 1A, the increased activation of proMMP-2 by co-culture was enhanced by EGF-treatment. The enhancement of proMMP-2 activation by EGF was also dose-dependent with a notable effect at 10 ng $ml^{-1}$ and the maximum at 100 ng $ml^{-1}$ (Figure 1B). These results suggest that EGF may act as a promoter of the activation of proMMP-2 induced by co-culture of A431 cells and human fibroblasts.

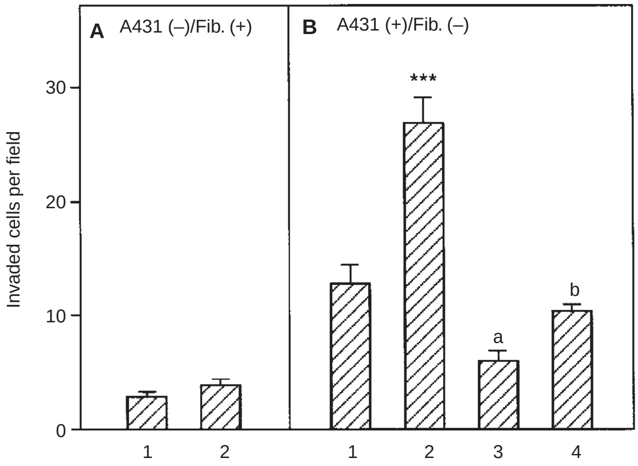

**Figure 2** Preferential invasion of A431 cells in co-culture. CFSE-labelled human dermal fibroblasts [Fib. (+)] at the 22nd passage and A431 cells [A431 (+)] at the 24th passage were added into the inserts together with CFSE-unlabelled A431 cells [A431 (–)] (**A**) and human dermal fibroblasts [Fib. (–)] (**B**), respectively, and then treated with or without EGF (100 ng $ml^{-1}$) in the presence or the absence of TIMP-2 (100 ng $ml^{-1}$) for 48 h as described in Materials and Methods. The number of fluoresced cells on the lower side in ten randomly chosen areas per membrane was counted under a fluorescent microscope at 200-fold magnification. The data were indicated as the mean ± s.d. of ten individual areas. Student's *t*-test was used for analysis of the data. ***, significantly differed from untreated co-culture ($P < 0.001$). a and b, significantly differed from untreated and EGF-treated co-culture respectively ($P < 0.001$). Lane 1, untreated co-culture; lane 2, EGF-treated co-culture; lane 3, TIMP-2-treated co-culture; lane 4, EGF and TIMP-2-treated co-culture

### Augmented proMMP-2 activation increases tumour invasion in co-culture

To investigate whether the augmentation of proMMP-2 activation causes the increase of tumour invasiveness in co-culture and further characterize which cell line invades, tumour invasion assay

© 1999 Cancer Research Campaign 

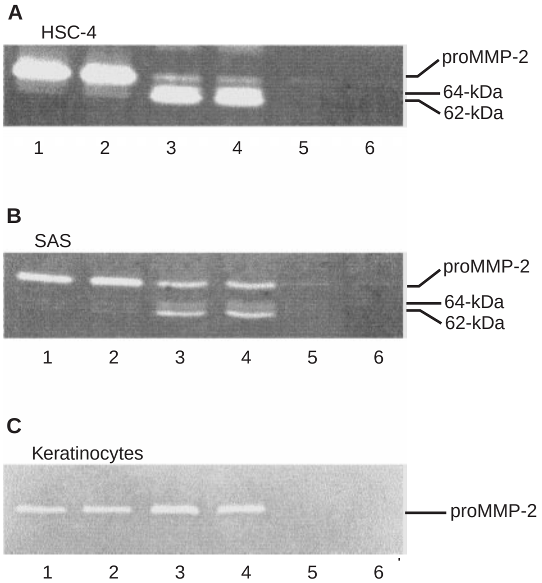

**Figure 3** Activation of proMMP-2 by co-culture of human dermal fibroblasts and human carcinoma cells or human keratinocytes. Confluent human dermal fibroblasts at the 16th passage were co-cultured with HSC-4 (**A**), SAS (**B**) or human keratinocytes at the 8th passage (**C**) at the cellular ratio of 4 for 24 h and then treated with or without EGF (100 ng ml$^{-1}$) for a further 24 h. An aliquot (10 μl) of the harvested culture medium was subjected to gelatin zymography as described in Materials and Methods. Three independent experiments were reproducible and typical data are shown. Lanes 1 and 2, fibroblasts alone; lanes 3 and 4, fibroblasts co-cultured with HSC-4, SAS or keratinocytes; lanes 5 and 6, HSC-4, SAS or keratinocytes alone. Cells in lanes 2, 4 and 6 were also treated with EGF (100 ng ml$^{-1}$)

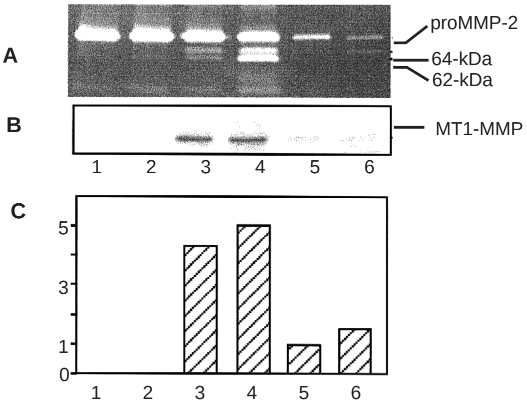

**Figure 4** Induction of MT1-MMP production in co-culture of A431 cells and human dermal fibroblasts. Confluent human dermal fibroblasts at the 13th passage were co-cultured with A431 cells at the 15th passage at the cellular ratio of 4 for 24 h. In monolayer culture of A431 cells, the number of cells seeded corresponded to that for the cellular ratio of 4. Those cells were treated with or without EGF (100 ng ml$^{-1}$) for another 24 h. The harvested culture medium and membrane fractions were subjected to gelatin zymography (**A**) and Western blot analysis (**B**), respectively, as described in Materials and Methods. The relative production of MT1-MMP was quantified by densitometric scanning and taking the control value of 1 in A431 cells alone (**C**). Three independent experiments were reproducible and typical data are shown. Lanes 1 and 2, fibroblasts alone; lanes 3 and 4, fibroblasts co-cultured with A431 cells; lanes 5 and 6, A431 cells alone. Cells in lanes 2, 4 and 6 were also treated with EGF (100 ng ml$^{-1}$)

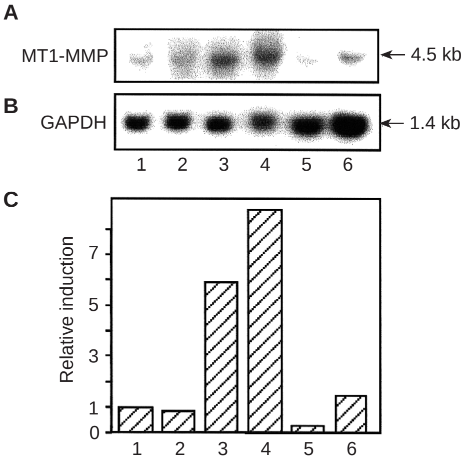

**Figure 5** Induction of MT1-MMP mRNA expression in co-culture of A431 cells and human dermal fibroblasts. Total RNA (15 μg) was subjected to Northern blot analysis as described in Materials and Methods. (**A**) MT1-MMP mRNA and (**B**) GAPDH mRNA. The relative amount of MT1-MMP mRNA was quantified by densitometric scanning and taking the control value of 1 in human dermal fibroblasts by standardizing with that of GAPDH mRNA (**C**). Three independent experiments were reproducible and typical data are shown. Lanes 1 and 2, fibroblasts alone; lanes 3 and 4, fibroblasts co-cultured with A431 cells; lanes 5 and 6, A431 cells alone. Cells in lanes 2, 4 and 6 were treated with EGF (100 ng ml$^{-1}$)

with a Matrigel model was performed. When A431 cells were co-cultured with fluoresced human fibroblasts, there was little number of fluorescence-positive cells detected regardless of the presence or the absence of EGF (100 ng ml$^{-1}$) (Figure 2A). On the other hand, when fluoresced A431 cells were co-cultured with human fibroblasts, the number of fluorescence-positive invaded cells was significantly increased (Figure 2B) (1.9-fold increase as compared with the fluoresced A431 cells alone, data not shown). EGF further augmented the invasion of fluorescence-positive cells in co-culture (1.8-fold), indicating that A431 cells but not human fibroblasts in co-culture preferentially invaded through Matrigel. In addition, the increased invasive activity of A431 cells was inhibited by exogenously adding TIMP-2 (55 and 61% inhibition in EGF-untreated and -treated co-culture, respectively). These results suggest that the advanced proMMP-2 activation by co-culture of A431 cells and human fibroblasts substantially increases tumour invasiveness.

### Human carcinoma cells but not normal human keratinocytes augment proMMP-2 activation in human fibroblasts

To examine whether the enhancement of proMMP-2 activation was specifically caused by cell–cell interaction between tumour cells and normal cells, the co-culture was performed with other human squamous carcinoma cell lines, HSC-4 and SAS, and normal human keratinocytes. As shown in Figure 3, no proMMP-2 was detected in these carcinoma cells or human keratinocytes (lane 5). Intense activation of proMMP-2 was observed in fibroblasts co-cultured with HSC-4 or SAS, but not with human normal keratinocytes (lane 3). Unlike A431 cells, EGF (100 ng ml$^{-1}$) did not enhance the activation of proMMP-2 in the co-culture of HSC-4 or SAS (lane 4) because proMMP-2 activation by the co-culture was mostly completed. Therefore, we suggest that the augmentation of proMMP-2 activation is specifically occurred in the co-culture of human squamous carcinoma cells and normal human dermal fibroblasts.

 *© 1999 Cancer Research Campaign*

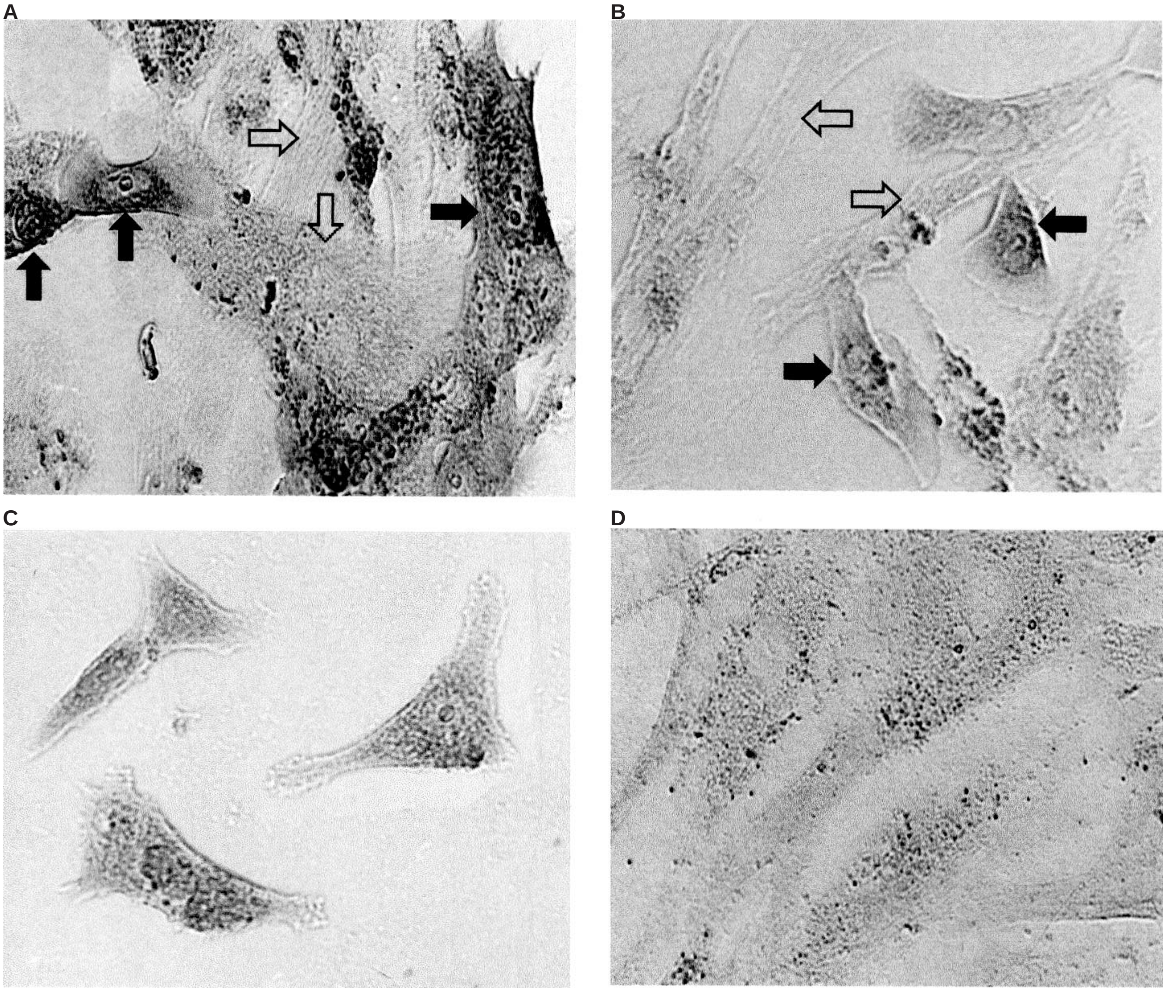

**Figure 6** The production of MT1-MMP is augmented in co-cultured A431 cells, but not fibroblasts. Human dermal fibroblasts at the 19th passage were co-cultured with A431 cells at the 20th passage at the cellular ratio of 4 for 24 h. (**A** and **B**) Co-culture stained with monoclonal anti-(human MT1-MMP) antibody and non-immune mouse IgG, respectively. (**C** and **D**) A431 cells and human dermal fibroblasts stained with monoclonal anti-(human MT1-MMP) antibody, respectively. Closed and open arrows in Panels **A** and **B** indicate A431 cells and fibroblasts respectively

### Co-culture induces the production of MT1-MMP along with the augmentation of its gene expression

Next we investigated whether the augmentation of proMMP-2 activation was related to an increase in the production of MT1-MMP. Although MT1-MMP was not detected in fibroblasts and only slightly in A431 cells, the co-culture enhanced MT1-MMP production (Figure 4B, lane 3; 4.3-fold vs A431 cell alone) with consequent activation of proMMP-2 (Figure 4A, lane 3). Northern blot analysis (Figure 5) also indicated that cell–cell interaction enhanced the expression of MT1-MMP mRNA as compared to that for fibroblasts and A431 cells (5.8-fold and 19.3-fold, respectively). A similar increase in MT1-MMP mRNA was observed in fibroblasts cultured with HSC-4 or SAS (data not shown), indicating that the augmentation of MT1-MMP production by co-culture resulted from increasing its gene expression. On the other hand, EGF further augmented the co-culture-mediated production of MT1-MMP and its mRNA (1.2- and 1.4-fold, respectively) and concomitantly enhanced proMMP-2 activation (Figures 4 and 5, lane 4). These results suggest that EGF augments the production of MT1-MMP, and thereby the activation of proMMP-2 is increased in the co-culture of A431 cells and human dermal fibroblasts.

### Increased production of MT1-MMP in A431 cells, but not in human fibroblasts of co-culture

We further characterized which cell line expressed MT1-MMP when A431 cells and fibroblasts were co-cultured. As shown in Figure 6A, the expression of MT1-MMP was significantly augmented in A431 cells, but not in human fibroblasts in the co-culture. By contrast, when the cells were stained by non-immune mouse IgG, the signals of MT1-MMP were no longer detected in A431 cells in the co-culture (Figure 6B). On the other hand, MT1-MMP was not detected in either monolayer culture of A431 cells or human fibroblasts (Figure 6C and D, respectively). These results indicated that MT1-MMP in tumour cells was preferentially produced by cell–cell interaction and suggested that the sequential activation of proMMP-2 by MT1-MMP may progress on the tumour cell surface.

### DISCUSSION

The increased production of proMMP-2 is known to relate to tumour invasiveness both in vivo and in vitro (Pyke et al, 1992; Stearns and Wang, 1993; Stetler-Stevenson et al, 1993). Azzam et

© 1999 Cancer Research Campaign al (1993) reported that increased activation of proMMP-2 is restricted to highly invasive human breast cancer cells, but it is not fully understood how the activation of proMMP-2 is accomplished in cancer cells or tissue. In the present study, we demonstrated that human squamous carcinoma cells interacted with normal human fibroblasts augmented the activation of proMMP-2, whereas normal human keratinocytes did not, and further that tumour cells in the co-culture preferentially invaded through Matrigel in in vitro tumour invasion assay system. These observations propose an in vivo mechanism in which the increased activity of MMP-2 associated with tumour invasiveness is evoked by the cell–cell interaction between the grown tumour cells and the surrounding normal cells.

MT-MMPs have been cloned and characterized as a specific proMMP-2 activator. Sato et al (1994) demonstrated that MT1-MMP overexpressed in HT-1080 cells and NIH3T3 cells converts 72-kDa proMMP-2 into a 62-kDa active form, which is correlated with in vitro invasiveness of those cells. Pei and Weiss (1996) reported that a soluble mutant of MT1-MMP deleting the transmembrane domain proteolytically converts proMMP-2 to the active form. Since many investigators have focused on the molecular mechanism of proMMP-2 activation by MT-MMPs, there is little evidence concerning the regulation of MT-MMP production. Lohi et al (1996) reported that phorbol ester transcriptionally induces the production of MT1-MMP in HT-1080 cells and human fibroblasts. Our present study showed that co-culture of A431 cells and human fibroblasts increased the production of MT1-MMP together with the augmentation of its mRNA level. A similar increase in MT1-MMP mRNA was observed in other types of carcinoma cells, HSC-4 and SAS co-cultured with human fibroblasts (data not shown). Thus, these results provide strong evidence that cell–cell interaction between carcinoma and normal stroma cells has the initiative in increasing the production of MT1-MMP.

Recent studies have shown the mechanism by which cell–cell interaction influences proMMPs expression in various cell types. Ito et al (1995) reported that breast adenocarcinoma MCF-7 cell-derived membrane bound factor together with the soluble one augments the production of proMMPs-1 and -3 and their inhibitor TIMP-1 in neighbouring stroma cells. Westerlund et al (1997) also reported that normal fibroblasts stimulate human ovarian cancer cells to increase their invasiveness and MMP-2 production in an autocrine and paracrine manner. We, however, demonstrated that both the membrane fraction and conditioned medium of A431 cells or fibroblasts modify neither the activation of proMMP-2 nor its production in each monolayer culture (data not shown). This result suggests that both A431 cells and human dermal fibroblasts in the monolayer culture do not produce any factors for increasing the activation of proMMP-2. Alternatively, it is possible that factor(s) expressed substantially in the co-culture condition participates in augmenting the activation of proMMP-2 as well as the production of MT1-MMP, although a principal mechanism by which proMMP-2 activation and MT1-MMP production are enhanced in this co-culture system tested remains unclear.

It has been reported that in host fibroblasts the gene expression of MT1-MMP is enhanced by soluble factor(s) secreted by invasive breast tumour cells and thereby the activation of proMMP-2 is occurred on the cell surface of fibroblasts (Polette et al, 1997). In contrast, our result by immunohistochemistry showed that MT1-MMP production in the co-culture was augmented predominantly in A431 cells but not in human fibroblasts, suggesting that the increase of proMMP-2 activation occurs on the tumour cell surface. In regard of the different observation, we postulate that proMMP-2 activation which is accomplished on the cell surface of either tumour or adjacent stroma cells may be dependent on the tumour cell type.

Our results showed that EGF further enhanced the activation of proMMP-2 in co-culture of A431 cells and fibroblasts. We also demonstrated that EGF induced the predominant invasion of A431 cells in the co-culture and the increased invasive activity was inhibited by exogenously adding TIMP-2. Taken together with our immunohistochemical evidence (Figure 6) and a previous paper (Singletary et al, 1987) these results suggest that in human carcinomas, at least A431 cells, EGF is very likely to be an enhancer of tumour invasiveness by increasing proMMP-2 activation on tumour cell surface.

We observed discrepancies in that the extent of proMMP-2 activation by the co-culture was low although the induction of MT1-MMP was quite high, and that EGF relatively elicited a slight enhancement of MT1-MMP gene and protein levels, but apparently augmented the activation of proMMP-2 as compared to the EGF-untreated co-culture. Concerning these points, recent studies indicating that TIMP-2 participates in the regulation of proMMP-2 activation by MT1-MMP are of particular interest. Will et al (1996) and Kinoshita et al (1996) reported that TIMP-2 preferentially inhibits MT1-MMP activity to decrease proMMP-2 activation. On the other hand, TIMP-2 has been shown to promote proMMP-2 activation by MT1-MMP (Strongin et al, 1995; Kinoshita et al, 1998). We preliminarily demonstrated that the production of TIMP-2 was increased by the co-culture and EGF decreased the augmented TIMP-2 production (T Sato et al, unpublished data). Thus, we speculate that the increased TIMP-2 production in co-culture may suppress the activation of proMMP-2 by MT1-MMP even though MT1-MMP production is augmented, and that the down-regulation of TIMP-2 production by EGF may subsequently accelerate the proMMP-2 activation in the co-culture. Otherwise, it is suggested that EGF may promote the TIMP-2-mediated ternary complex formation with MT1-MMP and proMMP-2 on cell surface to accomplish proMMP-2 activation in the co-culture. Further experiments will be required for addressing the involvement of TIMP-2 in MT1-MMP-mediated proMMP-2 activation in this co-culture system.

In conclusion, we demonstrated that direct attachment of carcinoma cells to human fibroblasts augments the production of MT1-MMP and consequently activates proMMP-2 on the tumour cell surface. Therefore, this possible mechanism plays an important role in the tumour invasion in vivo by increasing proteolytic activity to degrade extracellular matrices at the tumour–stroma junction.

### ACKNOWLEDGEMENTS

We thank Dr H Nagase for providing recombinant human TIMP-2 and R Kubota for experimental assistance.

### REFERENCES

Azzam H, Arand G, Lippman ME and Thompson EW (1993) Association of MMP-2 activation potential with metastatic progression in human breast cancer cell lines independent of MMP-2 production. *J Natl Cancer Inst* **85**: 1758–1764

Baramova EN, Bajou K, Remacle A, L'Hoir C, Krell HW, Weidle UH, Noel A and Foidart JM (1997) Involvement of PA/plasmin system in the processing of proMMP-9 and in the second step of pro-MMP-2 activation. *FEBS Lett* **405**: 157–162

 *© 1999 Cancer Research Campaign*

Birkedal-Hansen H, Moore WGI, Bodden MK, Windsor LJ, Birkedal-Hansen B, DeCarlo A and Engler JA (1993) Matrix metalloproteinases: a review. *Crit Rev Oral Biol Med* **4**: 197–250

Bronner-Fraser M (1985) Alterations in neural crest migration by a monoclonal antibody that affects cell adhesion. *J Cell Biol* **101**: 610–617

Ito A and Nagase H (1988) Evidence that human rheumatoid synovial matrix metalloproteinase 3 is an endogenous activator of procollagenase. *Arch Biochem Biophys* **267**: 211–2216

Ito A, Itoh Y, Sato T, Mori Y, Suzuki K and Nagase H (1991) Identification of rabbit uterine cervical procollagenase activator as rabbit matrix metalloproteinase 3 (stromelysin). *Comp Biochem Physiol* **99B**: 381–385

Ito A, Nakajima S, Sasaguri Y, Nagase H and Mori Y (1995) Co-culture of human breast adenocarcinoma MCF-7 cells and human dermal fibroblasts enhances the production of matrix metalloproteinases 1, 2 and 3 in fibroblasts. *Br J Cancer* **71**: 1039–1045

Ito A, Yamada M, Sato T, Sanekata K, Sato H, Seiki M, Nagase H and Mori Y (1998) Calmodulin antagonists increase the expression of membrane-type-1 matrix metalloproteinase in human uterine cervical fibroblasts. *Eur J Biochem* **251**: 353–358

Itoh Y, Binner S and Nagase H (1995) Steps involved in activation of the complex of promatrix metalloproteinase 2 (progelatinase A) and tissue inhibitor of metalloproteinases (TIMP)-2 by 4-aminophenylmercuric acetate. *Biochem J* **308**: 645–651

Kinoshita T, Sato H, Takino T, Itoh M, Akizawa T and Seiki M (1996) Processing of a precursor of 72-kilodalton type IV collagenase/gelatinase A by a recombinant membrane-type 1 matrix metalloproteinase. *Cancer Res* **56**: 2535–2538

Kinoshita T, Sato H, Okada A, Ohuchi E, Imai K, Okada Y and Seiki M (1998) TIMP-2 promotes activation of progelatinase A by membrane-type 1 matrix metalloproteinase immobilized on agarose beads. *J Biol Chem* **273**: 16098–16103

Laemmli UK (1970) Cleavage of structural proteins during the assembly of the head of bacteriophage T4. *Nature (London)* **227**: 680–685

Lohi J and Keski-Oja J (1995) Calcium ionophores decrease pericellular gelatinolytic activity via inhibition of 92-kDa gelatinase expression and decrease of 72-kDa gelatinase activation. *J Biol Chem* **270**: 17602–17609

Lohi J, Lehti K, Westermarck J, Kähäri V-M and Keski-Oja J (1996) Regulation of membrane-type matrix metalloproteinase-1 expression by growth factors and phorbol 12-myristate 13-acetate. *Eur J Biochem* **239**: 239–247

Lowry OH, Rosebrough NJ, Lewis Farr A and Randall RJ (1951) Protein measurement with the Folin phenol reagent. *J Biol Chem* **193**: 265–275

Mazzieri R, Masiero L, Zanetta L, Monea S, Onisto M, Garbisa S and Mignatti P (1997) Control of type IV collagenase activity by components of the urokinase-plasmin system: a regulatory mechanism with cell-bound reactants. *EMBO J* **16**: 2319–2332

Ogata Y, Enghild JJ and Nagase H (1992) Matrix metalloproteinase 3 (stromelysin) activates the precursor for the human matrix metalloproteinase 9. *J Biol Chem* **267**: 3581–3584

Okada Y, Morodomi T, Enghild JJ, Suzuki K, Yasui A, Nakanishi I, Salvesen G and Nagase H (1990) Matrix metalloproteinase 2 from human rheumatoid synovial fibroblasts. Purification and activation of the precursor and enzymic properties. *Eur J Biochem* **194**: 721–730

Ozawa S, Ueda M, Ando N, Abe O and Shimizu N (1987) High incidence of EGF receptor hyperproduction in esophageal squamous-cell carcinomas. *Int J Cancer* **39**: 333–337

Pei D and Weiss SJ (1996) Transmembrane-deletion mutants of the membrane-type matrix metalloproteinase-1 process progelatinase A and express intrinsic matrix-degrading activity. *J Biol Chem* **271**: 9135–9140

Polette M, Gilles C, Marchard V, Seiki M, Tournier J-M and Birembaut P (1997) Induction of membrane-type matrix metalloproteinase 1 (MT1-MMP) expression in human fibroblasts by breast adenocarcinoma cells. *Clin Exp Metastasis* **15**: 157–163

Puente XS, Pendás AM, Llano E, Velasco G and López-Otín C (1996) Molecular cloning of a novel membrane-type matrix metalloproteinase from a human breast carcinoma. *Cancer Res* **56**: 944–949

Pyke C, Ralfkiær E, Huhtala P, Hurskainen T, Danø K and Tryggvason K (1992) Localization of messenger RNA for *M*r 72 000 and 92 000 type IV collagenase in human skin cancers by in situ hybridization. *Cancer Res* **52**: 1336–1341

Sato H, Takino T, Okada Y, Cao J, Shinagawa A, Yamamoto E and Seiki M (1994) A matrix metalloproteinase expressed on the surface of invasive tumor cells. *Nature* **370**: 61–65

Sato T, Kirimura Y and Mori Y (1997) The co-culture of dermal fibroblasts with human epidermal keratinocytes induces increased prostaglandin $E_2$ production and cyclooxygenase 2 activity in fibroblasts. *J Invest Dermatol* **109**: 334–339

Seltzer JL, Lee A-Y, Akers KT, Sudbeck B, Southon EA, Wayner EA and Eisen AZ (1994) Activation of 72-kDa type IV collagenase/gelatinase by normal fibroblasts in collagen lattices is mediated by integrin receptors but is not related to lattice contraction. *Exp Cell Res* **213**: 365–374

Shima I, Sasaguri Y, Kusukawa J, Nakano R, Yamana H, Fujita H, Kakegawa T and Morimatsu M (1993) Production of matrix metalloproteinase 9 (92-kDa gelatinase) by human oesophageal squamous cell carcinoma in response to epidermal growth factor. *Br J Cancer* **67**: 721–727

Singletary SE, Baker FL, Spitzer G, Tucker SL, Tomasovic B, Brock WA, Ajani JA and Kelly AM (1987) Biological effect of epidermal growth factor on the in vitro growth of human tumors. *Cancer Res* **47**: 403–406

Stearns ME and Wang M (1993) Type IV collagenase (*M*r 72 000) expression in human prostate: benign and malignant tissue. *Cancer Res* **53**: 878–883

Stetler-Stevenson WG, Aznavoorian S and Liotta LA (1993) Tumor cell interactions with the extracellular matrix during invasion and metastasis. *Annu Rev Cell Biol* **9**: 541–573

Strongin AY, Marmer BL, Grant GA and Goldberg GI (1993) Plasma membrane-dependent activation of the 72-kDa type IV collagenase is prevented by complex formation with TIMP-2. *J Biol Chem* **268**: 14033–14039

Strongin AY, Collier I, Bannikov G, Marmer BL, Grant GA and Goldberg GI (1995) Mechanism of cell surface activation of 72-kDa type IV collagenase: isolation of the activated form of the membrane metalloproteinase. *J Biol Chem* **270**: 5331–5338

Takahashi S, Ito A, Nagino M, Mori Y, Xie B and Nagase H (1991) Cyclic adenosine 3′, 5′-monophosphate suppresses interleukin 1-induced synthesis of matrix metalloproteinases but not of tissue inhibitor of metalloproteinases in human uterine cervical fibroblasts. *J Biol Chem* **266**: 19894–19899

Takino T, Sato H, Shinagawa A and Seiki M (1995) Identification of the second membrane-type matrix metalloproteinase (MT-MMP-2) gene from a human placenta cDNA library: MT-MMPs from a unique membrane-type subclass in the MMP family. *J Biol Chem* **270**: 23013–23020

Ueno H, Nakamura H, Inoue M, Imai K, Noguchi M, Sato H, Seiki M and Okada Y (1997) Expression and tissue localization of membrane-types 1, 2, and 3 matrix metalloproteinases in human invasive breast carcinomas. *Cancer Res* **57**: 2055–2060

Ward RV, Atkinson SJ, Slocombe PM, Docherty AJP, Reynolds JJ and Murphy G (1991) Tissue inhibitor of metalloproteinases-2 inhibits the activation of 72 kDa progelatinase by fibroblast membranes. *Biochim Biophys Acta* **1079**: 242–246

Westerlund A, Hujanen E, Puistola U and Turpeenniemi-Hujanen T (1997) Fibroblasts stimulate human ovarian cancer cell invasion and expression of 72-kDa gelatinase A (MMP-2). *Gynecol Oncol* **67**: 76–82

Will H and Hinzmann B (1995) cDNA sequence and mRNA distribution of a novel human matrix metalloproteinase with a potential transmembrane domain. *Eur J Biochem* **231**: 602–608

Will H, Atkinson SJ, Butler GS, Smith B and Murphy G (1996) The soluble catalytic domain of membrane type 1 matrix metalloproteinase cleaves the propeptide of progelatinase A and initiates autoproteolytic activation: regulation by TIMP-2 and TIMP-3. *J Biol Chem* **271**: 17119–17123

Yamamoto M, Mohanam S, Sawaya R, Fuller GN, Seiki M, Sato H, Gokaslan ZL, Liotta LA, Nicolson GL and Rao JS (1996) Differential expression of membrane-type matrix metalloproteinase and its correlation with gelatinase A activation in human malignant brain tumors in vitro and in vivo. *Cancer Res* **56**: 384–392

© 1999 Cancer Research Campaign 
